# Robot-assisted esophagectomy may improve perioperative outcome in patients with esophageal cancer – a single-center experience

**DOI:** 10.3389/fonc.2022.966321

**Published:** 2022-08-17

**Authors:** Johanna Betzler, Lea Elfinger, Sylvia Büttner, Christel Weiß, Nuh Rahbari, Alexander Betzler, Christoph Reißfelder, Mirko Otto, Susanne Blank, Sebastian Schölch

**Affiliations:** ^1^Department of Surgery, Universitätsmedizin Mannheim, Medical Faculty Mannheim, Heidelberg University, Mannheim, Germany; ^2^Junior Clinical Cooperation Unit Translational Surgical Oncology, German Cancer Research Center (DKFZ), Heidelberg, Germany; ^3^German Cancer Research Center (DKFZ) - Hector Cancer Institute at University Medical Center Mannheim, Mannheim, Germany; ^4^Department of Medical Statistics, Biomathematics and Information Processing, Medical Faculty Mannheim, Heidelberg University, Mannheim, Germany

**Keywords:** minimally invasive esophagectomy, esophageal surgery, abdominothoracic esophagectomy, robotic surgery, DaVinci

## Abstract

**Background:**

Although the introduction of minimally invasive surgical techniques has improved surgical outcomes in recent decades, esophagectomy for esophageal cancer is still associated with severe complications and a high mortality rate. Robot-assisted surgery is already established in certain fields and robot-assisted esophagectomy may be a possible alternative to the standard minimally invasive esophagectomy. The goal of this study was to investigate whether robot assistance in esophagectomy can improve patient outcome while maintaining good oncological control.

**Material and methods:**

Data of all patients who underwent minimally invasive esophagectomy between January 2018 and November 2021 at University Hospital Mannheim was collected retrospectively. Patients were divided into two cohorts according to operative technique (standard minimally invasive (MIE) vs. robot-assisted esophagectomy (RAMIE), and their outcomes compared. In a separate analysis, patients were propensity score matched according to age, gender and histological diagnosis, leading to 20 matching pairs.

**Results:**

95 patients were included in this study. Of those, 71 patients underwent robot-assisted esophagectomy and 24 patients underwent standard minimally invasive esophagectomy. Robot-assisted esophagectomy showed a lower incidence of general postoperative complications (52.1% vs. 79.2%, p=0.0198), surgical complications (42.3% vs. 75.0%, p=0.0055), a lower rate of anastomotic leakage (21.1% vs. 50.0%, p=0.0067), a lower Comprehensive Complication Index (median of 20.9 vs. 38.6, p=0.0065) as well as a shorter duration of hospital stay (median of 15 vs. 26 days, p=0.0012) and stay in the intensive care unit (median of 4 vs. 7 days, p=0.028) than standard minimally invasive surgery. After additionally matching RAMIE and MIE patients according to age, gender and diagnosis, we found significant improvement in the RAMIE group compared to the MIE group regarding the Comprehensive Complication Index (median of 20.9 vs. 38.6, p=0.0276), anastomotic leakage (20% vs. 55%, p=0.0484) and severe toxicity during neoadjuvant treatment (0 patients vs. 9 patients, p=0.005).

**Conclusion:**

Robot-assisted surgery can significantly improve outcomes for patients with esophageal cancer. It may lead to a shorter hospital stay as well as lower rates of complications, including anastomotic leakage.

## Introduction

Esophageal cancer is the eighth most common cancer worldwide and an aggressive disease with a poor prognosis. The incidence of esophageal adenocarcinoma has been increasing especially in Western countries over the past decades as the incidence of risk factors such as obesity and gastroesophageal reflux disease has been rising rapidly (1, 2). At the time of diagnosis, more than 50% of patients present with unresectable or metastatic disease (3). This leads to poor 5-year overall survival rates of around 20%; in metastatic disease under 5% (4).

Outside of very early tumor stages, multimodal therapy has been established as a gold standard, including radiochemotherapy or chemotherapy, as well as surgical resection of the esophagus with the goal of complete tumor removal. Conventional (open), abdominothoracic esophagectomy was the first established technique for resection and often allows complete removal of the tumor, albeit with a high morbidity rate, most prominently anastomotic leakage and pulmonary complications secondary to thoracotomy (5).

In order to reduce complication rates and facilitate postoperative recovery, minimally invasive esophagectomy (MIE) was introduced. When compared to conventional esophagectomy, MIE results in lower blood loss, a lower rate of postoperative complications and perioperative morbidity in general, as well as improved quality of life (2, 6, 7). However, the higher cost of MIE and protracted learning curve as well as its technical complexity are obstacles in its implementation.

**Figure 1:**
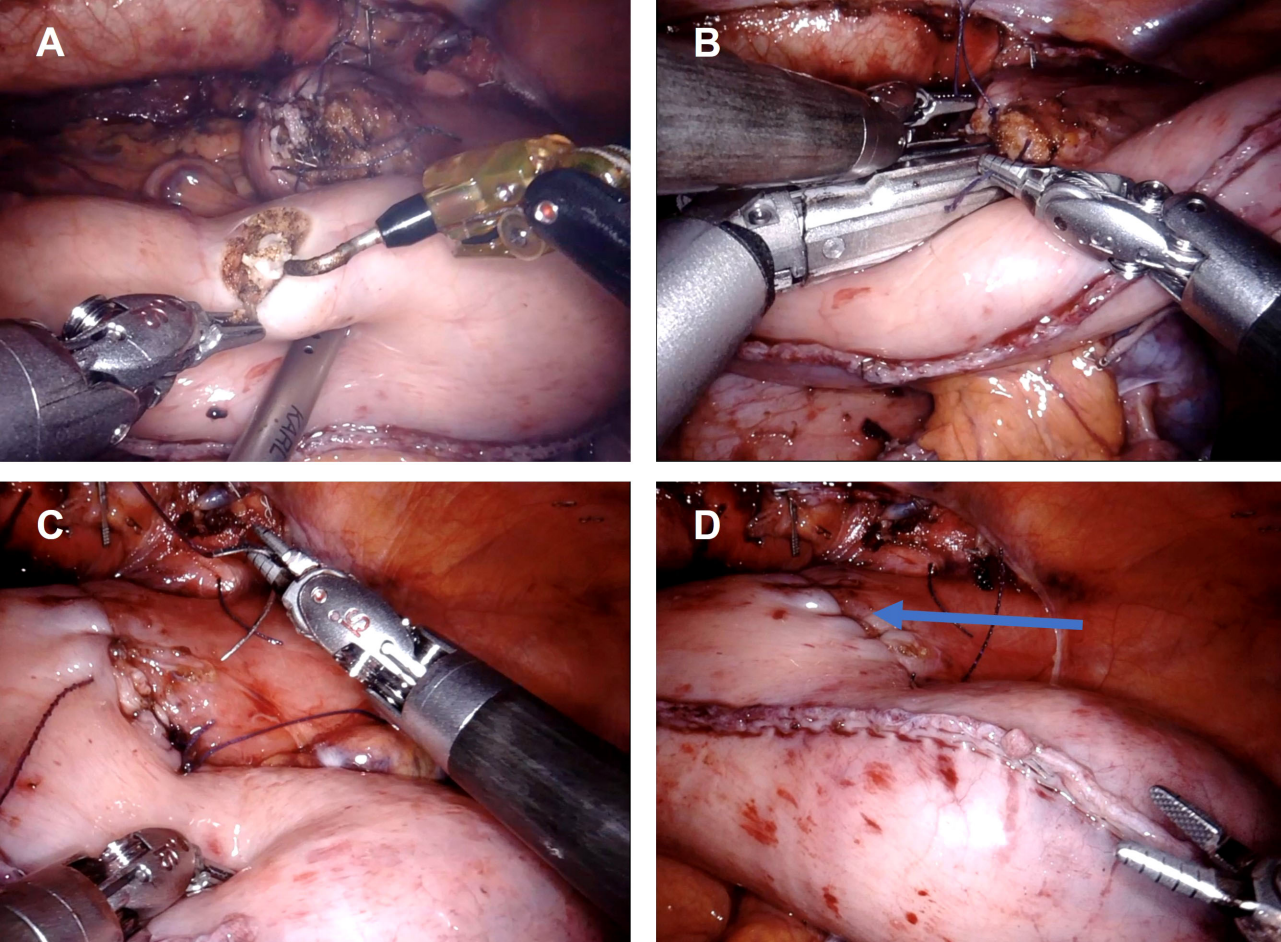
Robot-assisted linear stapled side-to-side esophagogastrostomy. **(A)** Opening of the gastric conduit. **(B)** Intrathoracic stapling of the anastomosis with the linear stapler. **(C)** Closure of the incision hole in esophagus and gastric conduit. **(D)** Completed side-to-side esophagogastrostomy (blue arrow indicates location of the esophagogastric anastomosis).

**Figure 2:**
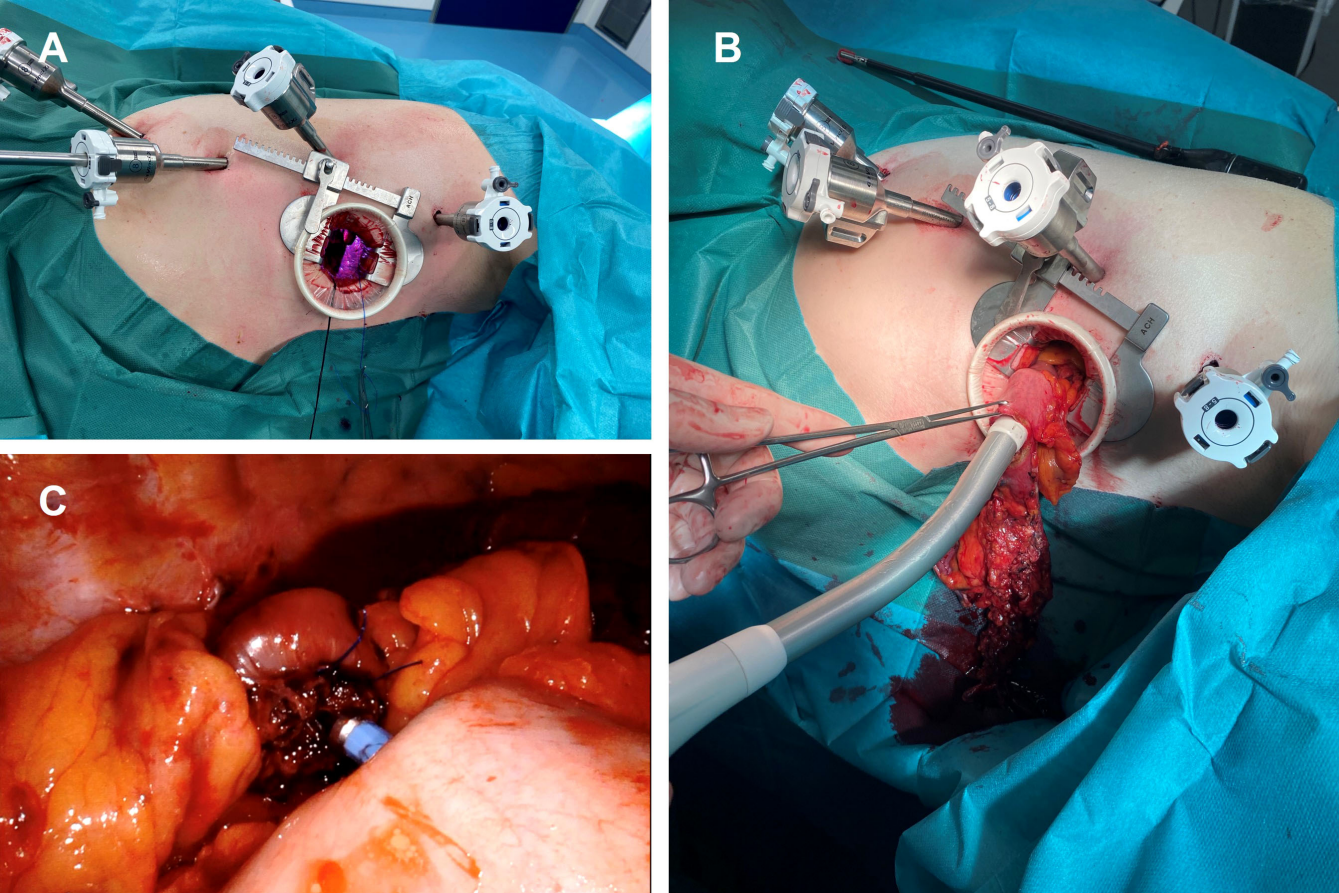
Robot-assisted circular stapled end-to-side esophagogastrostomy. **(A)** Thoracic port placement and small thoracotomy. **(B)** Insertion of the circular stapler into the gastric conduit. **(C)** Intrathoracic end-to-side esophagogastrostomy.

Recently, robot-assisted minimally invasive esophagectomy (RAMIE) has been introduced with the prospect of overcoming the technical limitations associated with MIE while maintaining good oncological outcomes (8). During RAMIE, the surgeon operates the robotic arms positioned at the patient from a console and benefits from an enlarged three-dimensional view of the operating field, a higher degree of freedom with the articulated instruments and stabilization of the naturally occurring tremor. Both the thoracoscopic and the laparoscopic parts of the procedure can be performed robotically, though in this study, we focused on the thoracoscopic part (esophagectomy, lymphadenectomy and esophagogastrostomy) while the abdominal part (formation of the gastric conduit) were performed as non-robotic laparoscopy (**Figures 1**, **2**).

Although evidence comparing RAMIE to standard MIE is still limited, RAMIE has shown a lower blood loss as well as a shorter intensive care unit (ICU) stay while maintaining high rates of R0 resections when compared to MIE (8–10). A previously conducted systematic review also found that RAMIE provided similar short-term mortality rates (11). The need for specialized training in handling the robot as well as significant financial requirements for the acquisition and maintenance of robotic systems have however been holding back many hospitals from routinely using RAMIE.

In early 2018, RAMIE was introduced in the Department of Surgery of University Hospital Mannheim as an alternative to the standard minimally invasive surgery, both for tumors of the esophagus and the esophagogastric junction regardless of histologic subtype.

In this study, we report our experiences with robotic resection of esophageal cancer and investigate whether use of a surgical robot can improve patient outcome and reduce postoperative complications.

## Material and methods

### Patient selection and study design

A retrospective database was built containing all patients who underwent minimally invasive surgery for esophageal cancer or cancer of the esophagogastric junction (AEG) with curative intent between January 2018 and November 2021 (n=95). Patients who underwent laparotomy (n=9) or a combination of open and minimally invasive esophagectomy (n=8) were excluded from the study, as well as patients who received a two-stage surgical procedure (n=4) and patients with preoperatively diagnosed metastatic disease. In one patient liver metastases were detected during surgery.

Patients with Siewert type III adenocarcinoma of the esophagogastric junction (i.e. proximal gastric cancer not involving the esophagogastric junction), were not included in this study and treated following the gastric cancer protocol.

The study focused on postoperative complications as well as length of surgery, intraoperative blood loss, length of ICU and length of hospital stay.

This study was reviewed and approved by the ethics board II of Heidelberg University prior to its initiation (approval number 2020-803R).

### Preoperative diagnostics and treatment

The tumor-node-metastasis (TNM) system was used for staging, which included endoscopy, endoscopic ultrasonography and a computer tomography (CT) scan of chest and abdomen. Only in cases in which the CT results suggested lymph node metastases not included in the standard lymphadenectomy or which would otherwise change the therapeutic strategy, a PET (positron emission tomography)/CT scan was performed. Biopsies were taken during endoscopy to determine histopathologic subtype and grading of the tumor. A multidisciplinary board discussed every case prior to treatment initiation.

Patient weight was recorded before neoadjuvant therapy and again on the day before surgery. The difference between those weights constituted the weight change during neoadjuvant therapy.

Preexisting conditions were classified into four categories: cardiovascular disease, pulmonary disease, diabetes and other malignancies besides esophageal cancer. Serum levels of albumin and cholesterol were routinely determined preoperatively and used as indicators for nutritional status (12–14).

Neoadjuvant therapy was given to patients with locally advanced tumors (cT3, cT4 or cN+) without the presence of distant metastases (cM0). Depending on tumor stage and histopathologic type, patients preoperatively received either chemotherapy according to the FLOT protocol (15) (adenocarcinoma) or radiochemotherapy according to CROSS protocol (16) (squamous cell carcinoma (SCC)).

Patients with early-stage tumors (cT1-2N0) did not receive neoadjuvant therapy. Toxicity of neoadjuvant therapy was estimated using the National Cancer Institute’s “Common Terminology Criteria for Adverse Effects” (CTCAE). We defined symptoms of grade three and higher, as well as all side effects that led to patient hospitalization, as severe toxicity (17, 18).

### Operative techniques

The availability of the robotic system on the day of surgery determined whether MIE or RAMIE was performed. The same three surgeons operated on all of the patients, two of which were in training during the study period.

All esophagectomies were performed with an Ivor-Lewis (right thoracic) approach and consisted of a laparoscopy and a thoracoscopy. All patients in this study received an esophageal resection with two-field mediastinal and abdominal lymphadenectomy (D2 lymphadenectomy), reconstruction was performed as a gastric conduit and either side-to-side esophagogastrostomy with linear stapling technique (**Figure 1**) or end-to-side esophagogastrostomy using circular stapling technique (**Figure 2**). The anastomotic technique was changed from side-to-side linear stapling to end-to-side circular stapling in 2020 due to promising results of end-to-side circular stapling concerning anastomotic leakage and perioperative outcomes in several recent studies (19, 20). The anastomosis was mostly located intrathoracically. Two patients in the MIE group had a cervical anastomosis. In the RAMIE group, the DaVinci Xi system (Intuitive Surgical Inc, Sunnyvale, CA) was either used for the thoracoscopic part (n=60) or for both the laparoscopic and thoracoscopic parts (n=11). Operating time included repositioning of the patient between the laparoscopic and thoracoscopic parts as well as docking and undocking of the surgical robot. Blood loss during surgery was approximated by operating room personnel. The resected specimen was evaluated during routine pathological work-up by board-certified pathologists of the Department of Pathology of University Hospital Mannheim.

### Postoperative management and complications

All patients were postoperatively transferred to either the intermediate care ward (IMC) or the ICU depending on the respiratory state of the patient. In this study, IMC and ICU stay will be summarized under the data point “length of ICU-stay”.

Parenteral nutrition was used only when enteral food intake was not sufficient. All patients were encouraged to engage in physical activity according to their capabilities, starting on the day of surgery and supported by trained physical therapists.

Complications were categorized as either medical or surgical. Medical complications were divided into cardiac and pulmonary, the latter of which includes pneumonia. Under surgical complications, we summarized all complications that were related directly to the surgical procedure (e.g. postoperative hemorrhage). The incidence of anastomotic leakage as the most prominent surgical complication was analyzed separately.

If patients presented with elevated or increasing infectious parameters after postoperative day three (fever ≥ 38.5°C, leukocyte increase of ≥ 5,000/µl or total amount of ≥ 20,000/µl, CRP increase of ≥ 50mg/l or total amount of ≥ 200mg/l) an esophagogastroduoenoscopy (EGD) was performed. If no other reason was found for the elevated infectious parameters, prophylactic endoscopic vacuum therapy was started, even if no anastomotic insufficiency could be seen during EGD.

Postoperative complications are often ranked according to the Clavien-Dindo classification, a seven grade system in which higher grades indicate more severe complications (21). Usually only higher grades of complication (grades 2b or higher) in a patient are reported for statistical analysis, leading to an incorrect representation of the actual overall morbidity. To avoid this, we used the Comprehensive Complication Index (CCI) which is based on the Clavien-Dindo scores but includes all postoperative complications, weighted according to their severity, producing a score between 0 (no complications) and 100 (death) (22). An online calculator (https://www.assessurgery.com/) was used to calculate the CCI.

Follow-up was scheduled according to guidelines, with the first appointment two weeks after discharge.

### Propensity score matching

To reduce bias due to confounding variables, propensity score matching (PSM) was performed. PSM included age, sex and histopathological diagnosis of the tumor (AEG I, AEG II, SCC). The Greedy matching algorithm was used to form matched pairs between the 71 patients receiving RAMIE and the 24 patients receiving MIE. A caliper width (maximum allowable difference in propensity scores) of 0.25 was used.

### Statistical analysis

SAS statistical analysis software release 9.4 (SAS Institute Inc., Cary, NC, USA) was used for propensity score matching as well as other statistical analysis.

Qualitative variables were given as absolute and relative frequencies. Median and interquartile range (IQR) were calculated for non-normally distributed values. Mean and standard deviation (SD) were calculated for quantitative, normally distributed values.

Chi-square test was used for categorical variables. In cases of numbers lower than expected, Fisher’s exact test was performed. Normally distributed data were compared using the Student’s t-test. The Mann-Whitney-U-test was used for data not following a Gaussian distribution. All statistical tests comparing two groups were two-tailed. A *p*-value of <0.05 was considered statistically significant.

## Results

A total of 95 patients were included in this study; 71 patients (74.7%) underwent RAMIE, while 24 patients (25.3%) underwent standard MIE. In the RAMIE group, in 11 patients the surgical robot was used for both the thoracoscopic and laparoscopic part, in 60 patients the surgical robot was used only for the thoracoscopic part.

### Demographic and clinicopathological characteristics

The demographic data are presented in **Table 1**. The majority of patients (82.1%) were male with a mean age of 64 years. Most tumors were preoperatively identified as adenocarcinoma (AEG I in 43.2%, AEG II in 35.8%). In 60.0% of patients the tumor stage was identified as cT3 or higher, and in 68.4% of patients as cN+ (positive lymph node stage). The majority of patients (84.2%) received neoadjuvant therapy.

**Table 1:** Demographic and clinicopathological characteristics.

	Total	Type of surgery		*p*-value
	n=95	RAMIE (n=71)	MIE (n=24)	
**Sex**				0.5471
male	78 (82.1%)	57 (80.3%)	21 (87.5%)	
female	17 (17.9%)	14 (19.7%)	3 (12.5%)	
**Age, years** (mean, [SD])	64.1 [10.3]	63.2 [10.1]	66.7 [10.5]	0.1503
**Histopathology**				0.2502
AEG I	41 (43.2%)	34 (47.9%)	7 (29.2%)	
AEG II	34 (35.8%)	24 (33.8%)	10 (41.7%)	
SCC	20 (21.1%)	13 (18.3%)	7 (29.2%)	
**cT**				0.9046
1	10 (10.5%)	8 (11.3%)	2 (8.3%)	
2	14 (14.7%)	10 (14.1%)	4 (16.7%)	
3	55 (57.9%)	42 (59.1%)	13 (54.2%)	
4	2 (2.1%)	1 (1.4%)	1 (4.2%)	
x	14 (14.7%)	10 (14.1%)	4 (16.7%)	
**cN**				0.0945
+	65 (68.4%)	45 (63.4%)	20 (83.3%)	
0	20 (21.1%)	16 (22.5%)	4 (16.7%)	
x	10 (10.5%)	10 (14.1%)	0 (0.0%)	
**Neoadjuvant therapy**	80 (84.2%)	59 (83.1%)	21 (87.5%)	0.7535
Toxicity	39 (48.8%)	25 (42.4%)	14 (66.7%)	0.0759
Severe Toxicity	16 (42.1%)	6 (25%)	10 (71.4%)	**0.0077**
**Pre-existing conditions**				
Cardiovascular	55 (57.9%)	40 (56.3%)	15 (62.5%)	0.6401
Pulmonary	15 (15.8%)	11 (15.5%)	4 (16.7%)	1.0000
Diabetes	10 (10.5%)	7 (9.9%)	3 (12.5%)	0.7094
Other malignancies	14 (14.7%)	11 (15.5%)	3 (12.5%)	0.3018
**Nutritional status** Albumin, g/dl (median, [IQR])	37.4 [34.7-39.8]	37.6 [35.3-39.9]	37.0 [32.6-39.5]	0.1956
Cholesterol, mg/dl (mean, [SD])	209.7 [49.9]	211.3 [51.7]	204.4 [44.2]	0.8242
Preoperative BMI, kg/m^2^ (median, [IQR])	25.2 [22.6-27.9]	25.3 [22.8-27.9]	24.7 [22.0-27.6]	0. 6622

RAMIE, robot-assisted minimally invasive esophagectomy; MIE, minimally invasive esophagectomy; AEG, adenocarcinoma of the esophagogastric junction; SCC, squamous cell carcinoma; SD, standard deviation; IQR, interquartile range; p<0.05 are marked in bold.

Baseline characteristics of both groups were statistically compared to ensure similarity. The only significant difference between both groups was the report of severe toxicity during neoadjuvant therapy, which was higher in the MIE group (71.4% vs. 25%, p=0.0077).

The histopathological postoperative data are presented in **Table 2**. No significant differences between the two groups were found in (y)pT, (y)pN and (y)pM statuses, the number of resected lymph nodes, the resection status or the ratio of positive to total number of resected nodes.

**Table 2:** Histopathological data.

	Total	Type of surgery		*p*-value
	n=95	RAMIE (n=71)	MIE (n=24)	
**TNM Classification** **(y)pT**				0.1764
0	26 (27.4%)	23 (32.4%)	3 (12.5%)	
1	20 (21.1%)	15 (21.1%)	5 (20.8%)	
2	11 (11.6%)	9 (12.7%)	2 (8.3%)	
3	36 (37.9%)	23 (32.4%)	13 (54.2%)	
4	2 (2.1%)	1 (1.4%)	1 (4.2%)	
**(y)pN**				0.4317
0	59 (62.1%)	45 (63.4%)	14 (58.3%)	
1	23 (24.2%)	15 (21.1%)	8 (33.3%)	
2	10 (10.5%)	9 (12.7%)	1 (4.2%)	
3	3 (3.2%)	2 (2.8%)	1 (4.2%)	
**(y)pM**				0.4434
0	93 (97.9%)	70 (98.6%)	23 (95.8%)	
1	2 (2.1%)	1 (1.4%)	1 (4.2%)	
**R-Status**				0.3253
R0	90 (94.7%)	66 (93.0%)	24 (100%)	
R1	5 (5.3%)	5 (7.0%)	0 (0.0%)	
**Lymph nodes**				
Resected number (median, [IQR])	24 [19-34]	24.0 [19-34]	23.5 [18.5-32.3]	0.6342
Ratio of tumor affected to resected lymph nodes (median, [IQR])	0.0 [0.0-0.06]	0.0 [0.0-0.05]	0.0 [0.0-0.06]	0.7273

RAMIE, robot-assisted minimally invasive esophagectomy; MIE, minimally invasive esophagectomy; SD, standard deviation; IQR, interquartile range; p<0.05 are marked in bold.

### Perioperative data

Perioperative data of patients are presented in **Table 3**. There were no intraoperative complications.

**Table 3:** Perioperative data.

	Total	Type of surgery		*p*-value
	n=95	RAMIE (n=71)	MIE (n=24)	
**Characteristics of surgery** Length of surgery, minutes (median, [IQR])	395.0 [360.5-449.0]	395.0 [351.0-448.5]	399.5 [367.5-456.0]	0.6685
Blood loss, ml (median, [IQR])	275 []	250 [200-400]	400 [200-500]	0.1258
Blood transfusion	9 (9.9%)	5 (7.6%)	4 (17.4%)	0.2228
Anastomotic technique				**0.0406**
Linear stapling	63 (66.3%)	44 (62.0%)	19 (79.2%)	
Circular stapling	32 (33.7%)	27 (38.0%)	5 (20.8%)	
**Length of stay**, days				
ICU (median, [IQR])	4 [3-9]	4 [3-6.5]	7 [4-18.5]	**0.0280**
Total (median, [IQR])	17 [11-28]	15 [11-25.5]	26 [13.8-61]	**0.0012**
**Complications** Any complication	56 (59.0%)	37 (52.1%)	19 (79.2%)	** 0.0198**
**Clavien Dindo**				**0.0188**
0	39 (41.1%)	34 (47.9%)	5 (20.8%)	
1	1 (1.1%)	0 (0.0%)	1 (4.2%)	
2	10 (10.5%)	9 (12.7%)	1 (4.2%)	
3a + 3b	29 (30.5%)	19 (20.0%)	10 (41.7%)	
4a + 4b	12 (12.6%)	6 (6.3%)	6 (25.0%)	
5	4 (4.2%)	3 (4.2%)	1 (4.2%)	
**CCI** (median, [IQR])	20.9 [0-43.2]	20.9 [0-27.9]	38.6 [19.1-55.6]	**0.0065**
Surgical complications	48 (50.5%)	30 (42.3%)	18 (75.0%)	**0.0055**
** Anastomotic leakage**	27 (28.4%)	15 (21.1%)	12 (50.0%)	**0.0067**
Anastomotic technique				0.6885
Linear stapling	18 (18.9%)	10 (14.1%)	8 (33.3%)	
Circular stapling	9 (9.5%)	5 (7.0%)	4 (16.7%)	
Medical complications	31 (41.7%)	21 (29.6%)	10 (41.7%)	0.3181
Cardiac complications	16 (16.8%)	10 (14.1%)	6 (25.0%)	0.2228
Pulmonary complications	22 (23.2%)	15 (21.1%)	7 (29.2%)	0.4157

RAMIE, robot-assisted minimally invasive esophagectomy; MIE, minimally invasive esophagectomy; SD, standard deviation; IQR, interquartile range; ICU, intensive care unit; CCI, comprehensive complication index; p<0.05 are marked in bold.

The anastomotic techniques differed significantly between MIE and RAMIE (p=0.0406). Whereas 79% (n=19) of the patients in the MIE group received side-to-side linear stapling anastomosis, only 62% (n=44) in the RAMIE group received this anastomotic technique. 21% (n=5) of the MIE group received end-to-side circular stapling anastomosis, whereas patients in the RAMIE group received this anastomosis in 38% (n=27).

Median duration of surgery did not differ significantly between RAMIE and MIE (395.0 vs. 399.5 minutes, p=0.6685). Patients who received RAMIE had a shorter overall length of stay (median of 15 vs. 26 days, p=0.0012) and a shorter ICU stay (median of 4 vs. 7 days, p=0.0280). The rate of general postoperative complications was also lower in the RAMIE group (52.1% vs. 79.2%, p=0.0198) which reflects in a lower Clavien Dindo Score (p=0.0188) and a lower CCI (median of 20.9 vs. 38.6, p=0.0065). More specifically, the rate of surgical complications (42.3% vs. 75.0%, p=0.0055) and anastomotic leaks (21.1% vs. 50.0%, p=0.0067) were significantly lower in the RAMIE group. There were no significant differences in anastomotic leakage rates between the different anastomotic techniques when comparing RAMIE and MIE (p=0.6885). There was no difference in medical complications (46.2% vs. 47.4%, p=1.0000).

In the entire study population, two patients died within 30 days after surgery. This equals a 30-day-mortality rate of 2.1%.

### Propensity score matching

Patients in both groups were matched according to age, sex and histopathological diagnosis. The matched groups resulted in 20 pairs. Of these 40 patients, 36 (90.0%) were male, 14 (35.0%) had AEG type I tumors, 18 (45.0%) AEG type II and 8 (20.0%) SCC. The mean age of the RAMIE group was 64.4 and the mean age of the MIE group was 66.6.

In the preoperative data, we found a significant difference between the groups regarding the clinical lymph node stadium (cN) with 50.0% of patients in the RAMIE group vs. 85.0% of patients in the MIE group being staged as cN1 (p=0.0173). This did not reflect in the postoperative pathological staging (pN) which was not significantly different (p=0.8606). The difference in toxicity of neoadjuvant therapy was less pronounced after matching (42.9% vs. 68.4%, p=0.1420). Nevertheless, the rates of severe toxicity during neoadjuvant treatment remained significantly different between RAMIE and MIE (0 vs. 9 patients, p=0.0050). In the postoperative data, we found that the CCI (median of 20.9 vs. 38.6, p=0.0276) remained significantly lower in the RAMIE group after matching. Also, the rates of anastomotic leakage remained significantly lower in the RAMIE group (20% vs. 55%, p=0.0484). A non-significant tendency favouring RAMIE over MIE could be seen concerning overall postoperative complications (55.0% vs. 85.0%, p=0.0824) and surgical complications (50.0% vs. 80.0%, p=0.0958). ICU stay, overall hospital stay, Clavien Dindo Score, blood loss and other variables were not significantly different after matching.

## Discussion

The here presented findings suggest that robot-assisted surgery might positively influence the outcome of cancer patients undergoing esophageal resection. Patients who underwent RAMIE had a significantly shorter hospital stay and ICU stay, as well as a lower rate of postoperative complications as reflected in lower Clavien Dindo and CCI scores than patients who received standard MIE. More specifically, rates of surgical complications and anastomotic leaks were lower in the RAMIE group, although only anastomotic leakage rates remained significantly lower after matching. The procedure itself is safe with no intraoperative complications. Oncological results were comparable to MIE.

Several studies comparing RAMIE and MIE have found no significant difference in the report of postoperative complications (9, 23–27). In contrast, our study shows a lower incidence of overall and surgical complications in RAMIE and no difference in medical complications. This could be due to the technical advantages of a surgical robot when operating in narrow spaces such as the mediastinum. The anastomoses in both patient groups were mostly performed as thoracic anastomoses, which has been connected to a lower incidence of anastomotic leakage than cervical anastomoses (28). To eliminate bias as much as possible, we matched patients according to gender, age and diagnoses, using propensity score matching. Also, after matching the CCI and anastomotic leakage rates remained significantly lower in the RAMIE group.

A recently published retrospective and propensity score matched analysis by Babic et al. reported similar results comparing RAMIE and hybrid minimally invasive esophagectomy. Although the analyzed groups (RAMIE and hybrid surgery) were different to our study, Babic et al. also reported significantly shorter ICU stay and less complications in the RAMIE group. After propensity score matching, they could not find significant differences concerning anastomotic leakage rates, but a strong trend favoring RAMIE could be shown as well (29).

We found no significant difference in intraoperative blood loss which matches some previously published studies although there have been widely varying results concerning this topic in recent literature (26, 30). For the cohort in this study, the total length of hospital stay as well as ICU stay was significantly shorter in the RAMIE group. This might be due to reduced intra- and postoperative pain which could be achieved because the robotic arms are able to bend inside the chest and therefore produce less pressure on the ribs and the surrounding nerves (31). Intraoperatively reduced pain can lead to reduced stress of the patient and therefore might lead to better hemodynamic status during the procedure (32). The shortened ICU and hospitals stay is beneficial to the patient and reduces hospital expenditures, which in the long run could offset the higher cost of acquiring and maintaining a robotic system.

It is important to note that some studies include docking und undocking of the robot in the operating time while others do not, which leads to significant discrepancies in operating times (367 to 693 minutes) (33). The here presented operating times include the robot docking and undocking as well as repositioning of the patient for the thoracoscopic part of the procedure. The operating time in our RAMIE group is comparable to the operating times of other studies using a transthoracic approach and including the docking times of the robot into their operating times (34).

The existing evidence on the number of resected lymph nodes is ambivalent. Many studies have shown that RAMIE yields significantly higher numbers of resected lymph nodes than MIE (9, 26, 30, 35). Others have found no significant difference in number of lymph nodes resected between MIE and RAMIE, which reflects the findings in our study (8, 24, 25). This seems unsurprising, as the extent of lymphadenectomy between MIE and RAMIE is identical in our practice. Additionally, the influence of a more extensive lymph node resection on the oncological outcome is not clear. Park et al. reported no difference in 5-year survival rate between groups who received RAMIE and MIE, even though lymph node yield was higher in the RAMIE group (35).

RAMIE remains a technically challenging surgery that requires time and experience. It has been suggested that surgeons must perform between 20 and 70 robot-assisted operations to achieve proficiency (36). The cohort in this study has a relatively high rate of postoperative anastomotic leakage. During the trial period, two of the three surgeons performing the procedures were still in training for MIE and RAMIE, possibly explaining this increased rate of anastomotic leakage. Also, the anastomotic technique was changed in 2020 from linear side-to-side stapler anastomosis to end-to-side circular stapler anastomosis. Recently published results from the EsoBenchmark database indicate lower rates of anastomotic leakage for end-to-side circular stapler anastomoses (37). Despite these data, the change of anastomotic technique may have led to an even longer learning curve and therefore to higher complication rates. Matching this hypothesis, our analysis revealed no improvement of anastomotic leakage rate after introducing circular stapling anastomosis.

To increase the reliability of our results we performed propensity score matching. Unfortunately, the anastomotic technique could not be included into the matching as this resulted in too few matching pairs. As the comparison of anastomotic leakage between linear and circular stapling revealed no significant differences, the results of our analysis still can be interpreted as reliable.

The lower rate of anastomotic leakage in RAMIE indicates that the learning curve may be steeper in RAMIE and reflects the technical challenges of an intrathoracic, minimally invasive anastomosis without the increased flexibility of the robotic system. Another advantage of using a surgical robot is the two-person operating console that allows a learning surgeon to closely attend the surgical field and observe the technique of the operating surgeon.

This study has some limitations. Data were collected retrospectively. Surgery was not always performed by the same surgeon, which could directly influence the outcome, as different surgeons have different levels of surgical expertise and different learning curves.

Our analysis did not include long-term survival, which is an important factor when considering the surgical technique. Future research should focus on high-volume, multi-center, randomized, controlled trials comparing MIE to RAMIE, some of which are currently ongoing (38–40). Especially the currently recruiting ROBOT-2 trial might reveal interesting results in comparison to our study, as it is also conducted in German hospitals and especially the secondary outcome measures are comparable to the investigated variables of our cohort (40).

Our findings suggest that use of a robot-assisted surgery is safe and can positively impact the outcome of esophageal resection in esophageal cancer patients in terms of length of hospital stay and complications.

## Data availability statement

The raw data supporting the conclusions of this article will be made available by the authors, without undue reservation.

## Ethics statement

The studies involving human participants were reviewed and approved by Ethics Committee II, Medical Faculty Mannheim, Heidelberg University. The patients/participants provided their written informed consent to participate in this study.

## Author contributions

JB: study design, data collection and interpretation, writing and drafting of the manuscript. LE: data collection and interpretation, writing and drafting of the manuscript. SyB: statistical analysis, critical revision of manuscript. CW: statistical analysis, critical revision of manuscript. NR: data interpretation, critical revision of manuscript. AB: data interpretation, critical revision of manuscript. CR: study design, data interpretation, critical revision of manuscript. MO: study design, data interpretation, critical revision of manuscript. SB: study design, data collection and interpretation, critical revision of manuscript. SS: study design, data interpretation, critical revision of manuscript. All authors contributed to the article and approved the submitted version.

## Funding

SS is supported by Hector Stiftung II. JB is supported by Dieter Morszeck Foundation via the DKFZ Clinician Scientist Fellowship Program. For the publication fee we acknowledge financial support by "Deutsche Forschungsgemeinschaft" within the funding program "Open Access Publikationskosten" as well as by Heidelberg University.

## Acknowledgments

We thank the Department for Medical Statistics, Biomathematics and Information Processing of Medical Faculty Mannheim for their help with statistical data analysis.

## Conflict of interest

The authors declare that the research was conducted in the absence of any commercial or financial relationships that could be construed as a potential conflict of interest.

## Publisher’s note

All claims expressed in this article are solely those of the authors and do not necessarily represent those of their affiliated organizations, or those of the publisher, the editors and the reviewers. Any product that may be evaluated in this article, or claim that may be made by its manufacturer, is not guaranteed or endorsed by the publisher.

## References

[1] Lagergren J Bergström R Lindgren A Nyrén O. Symptomatic gastroesophageal reflux as a risk factor for esophageal adenocarcinoma. New Engl J Med (1999) 340:825–31. doi: 10.1056/NEJM199903183401101 10080844

[2] Pennathur A Gibson MK Jobe BA Luketich JD. Oesophageal carcinoma. Lancet (London England) (2013) 381:400–12. doi: 10.1016/S0140-6736(12)60643-6 23374478

[3] Enzinger PC Mayer RJ. Esophageal cancer. New Engl J Med (2003) 349:2241–52. doi: 10.1056/NEJMRA035010 14657432

[4] Mattiuzzi C Lippi G. Current cancer epidemiology. J Epidemiol Glob Health (2019) 9:217–22. doi: 10.2991/jegh.k.191008.001 PMC7310786 31854162

[5] Hulscher JBF van Sandick JW de Boer AGEM Wijnhoven BPL Tijssen JGP Fockens P. Extended transthoracic resection compared with limited transhiatal resection for adenocarcinoma of the esophagus. N Engl J Med (2002) 347:1662–9. doi: 10.1056/NEJMoa022343 12444180

[6] Biere SSAY van Berge Henegouwen MI Maas KW Bonavina L Rosman C Garcia JR. Minimally invasive versus open oesophagectomy for patients with oesophageal cancer: a multicentre, open-label, randomised controlled trial. Lancet (2012) 379:1887–92. doi: 10.1016/S0140-6736(12)60516-9 22552194

[7] van der Sluis PC van der Horst S May AM Schippers C Brosens LAA Joore HCA. Robot-assisted minimally invasive thoracolaparoscopic esophagectomy versus open transthoracic esophagectomy for resectable esophageal cancer: A randomized controlled trial. Ann Surg (2019) 269:621–30. doi: 10.1097/SLA.0000000000003031 30308612

[8] Jin D Yao L Yu J Liu R Guo T Yang K. Robotic-assisted minimally invasive esophagectomy versus the conventional minimally invasive one: A meta-analysis and systematic review. Int J Med Robot (2019) 15:e1988. doi: 10.1002/rcs.1988 30737881

[9] Tagkalos E Goense L Hoppe-Lotichius M Ruurda JP Babic B Hadzijusufovic E. Robot-assisted minimally invasive esophagectomy (RAMIE) compared to conventional minimally invasive esophagectomy (MIE) for esophageal cancer: a propensity-matched analysis. Dis Esophagus (2020) 33:doz060. doi: 10.1093/dote/doz060 31206577

[10] van der Sluis PC Ruurda JP Verhage RJJ van der Horst S Haverkamp L Siersema PD. Oncologic long-term results of robot-assisted minimally invasive thoraco-laparoscopic esophagectomy with two-field lymphadenectomy for esophageal cancer. Ann Surg Oncol (2015) 22 Suppl 3:S1350–1356. doi: 10.1245/s10434-015-4544-x PMC4686562 26023036

[11] Weksler B Sullivan JL. Survival after esophagectomy: A propensity-matched study of different surgical approaches. Ann Thorac Surg (2017) 104:1138–46. doi: 10.1016/j.athoracsur.2017.04.065 28760463

[12] van Stijn MFM Korkic-Halilovic I Bakker MSM van der Ploeg T van Leeuwen PAM Houdijk APJ. Preoperative nutrition status and postoperative outcome in elderly general surgery patients: a systematic review. JPEN J Parenter Enteral Nutr (2013) 37:37–43. doi: 10.1177/0148607112445900 22549764

[13] Aahlin EK Tranø G Johns N Horn A Søreide JA Fearon KC. Risk factors, complications and survival after upper abdominal surgery: A prospective cohort study. BMC Surg (2015) 15:83. doi: 10.1186/s12893-015-0069-2 26148685 PMC4494163

[14] Kuroda D Sawayama H Kurashige J Iwatsuki M Eto T Tokunaga R. Controlling nutritional status (CONUT) score is a prognostic marker for gastric cancer patients after curative resection. Gastric Cancer (2018) 21:204–12. doi: 10.1007/s10120-017-0744-3 28656485

[15] Al-Batran SE Homann N Pauligk C Goetze TO Meiler J Kasper S. Perioperative chemotherapy with fluorouracil plus leucovorin, oxaliplatin, and docetaxel versus fluorouracil or capecitabine plus cisplatin and epirubicin for locally advanced, resectable gastric or gastro-oesophageal junction adenocarcinoma (FLOT4): a randomised, phase 2/3 trial. Lancet (2019) 393:1948–57. doi: 10.1016/S0140-6736(18)32557-1 30982686

[16] van Hagen P Hulshof MCCM van Lanschot JJB Steyerberg EW van Berge Henegouwen MI Wijnhoven BPL. Preoperative chemoradiotherapy for esophageal or junctional cancer. N Engl J Med (2012) 366:2074–84. doi: 10.1056/NEJMoa1112088 22646630

[17] Arbuck SG. The revised common toxicity criteria: Version 2.0. CTEP website(1998). Available at: http://ctepinfonihgov.

[18] Trotti A Byhardt R Stetz J Gwede C Corn B Fu K. Common toxicity criteria: version 2.0. an improved reference for grading the acute effects of cancer treatment: impact on radiotherapy. Int J Radiat OncologyBiologyPhysics (2000) 47:13–47. doi: 10.1016/S0360-3016(99)00559-3 10758303

[19] Grimminger PP van der Horst S Ruurda JP van Det M Morel P van Hillegersberg R. Surgical robotics for esophageal cancer. Ann N Y Acad Sci (2018) 1434:21–6. doi: 10.1111/nyas.13676 29741233

[20] Tagkalos E van der Sluis PC Uzun E Berlth F Staubitz J Gockel I. The circular stapled esophagogastric anastomosis in esophagectomy: No differences in anastomotic insufficiency and stricture rates between the 25 mm and 28 mm circular stapler. J Gastrointest Surg (2021) 25:2242–9. doi: 10.1007/s11605-020-04895-x PMC8484169 33506342

[21] Dindo D Demartines N Clavien PA. Classification of surgical complications: A new proposal with evaluation in a cohort of 6336 patients and results of a survey. Ann Surg (2004) 240:205–13. doi: 10.1097/01.sla.0000133083.54934.ae PMC1360123 15273542

[22] Slankamenac K Graf R Barkun J Puhan MA Clavien PA. The comprehensive complication index: A novel continuous scale to measure surgical morbidity. Ann Surg (2013) 258:1–7. doi: 10.1097/SLA.0b013e318296c732 23728278

[23] Harbison GJ Vossler JD Yim NH Murayama KM. Outcomes of robotic versus non-robotic minimally-invasive esophagectomy for esophageal cancer: An American college of surgeons NSQIP database analysis. Am J Surg (2019) 218:1223–8. doi: 10.1016/j.amjsurg.2019.08.007 31500797

[24] Zhang Y Han Y Gan Q Xiang J Jin R Chen K. Early outcomes of robot-assisted versus thoracoscopic-assisted ivor Lewis esophagectomy for esophageal cancer: A propensity score-matched study. Ann Surg Oncol (2019) 26:1284–91. doi: 10.1245/s10434-019-07273-3 30843161

[25] Chen J Liu Q Zhang X Yang H Tan Z Lin Y. Comparisons of short-term outcomes between robot-assisted and thoraco-laparoscopic esophagectomy with extended two-field lymph node dissection for resectable thoracic esophageal squamous cell carcinoma. J Thorac Dis (2019) 11:3874–80. doi: 10.21037/jtd.2019.09.05 PMC6790445 31656660

[26] Deng H-Y Luo J Li S-X Li G Alai G Wang Y. Does robot-assisted minimally invasive esophagectomy really have the advantage of lymphadenectomy over video-assisted minimally invasive esophagectomy in treating esophageal squamous cell carcinoma? a propensity score-matched analysis based on short-term outcomes. Dis Esophagus (2019) 32:doy110. doi: 10.1093/dote/doy110 30496378

[27] Deng H-Y Huang W-X Li G Li S-X Luo J Alai G. Comparison of short-term outcomes between robot-assisted minimally invasive esophagectomy and video-assisted minimally invasive esophagectomy in treating middle thoracic esophageal cancer. Dis Esophagus (2018) 31(8). doi: 10.1093/dote/doy012 29538633

[28] Luketich JD Pennathur A Awais O Levy RM Keeley S Shende M. Outcomes after minimally invasive esophagectomy: review of over 1000 patients. Ann Surg (2012) 256:95–103. doi: 10.1097/SLA.0b013e3182590603 22668811 PMC4103614

[29] Babic B Müller DT Jung J-O Schiffmann LM Grisar P Schmidt T. Robot-assisted minimally invasive esophagectomy (RAMIE) vs. hybrid minimally invasive esophagectomy: propensity score matched short-term outcome analysis of a European high-volume center. Surg Endosc (2022). doi: 10.1007/s00464-022-09254-2 PMC9485091 35505259

[30] Li X-K Xu Y Zhou H Cong Z-Z Wu W-J Qiang Y. Does robot-assisted minimally invasive oesophagectomy have superiority over thoraco-laparoscopic minimally invasive oesophagectomy in lymph node dissection? Dis Esophagus (2021) 34:doaa050. doi: 10.1093/dote/doaa050 32582945

[31] Huang J Tian Y Li C Shen Y Li H Lv F. Robotic-assisted thoracic surgery reduces perioperative complications and achieves a similar long-term survival profile as posterolateral thoracotomy in clinical N2 stage non-small cell lung cancer patients: a multicenter, randomized, controlled trial. Transl Lung Cancer Res (2021) 10:4281–92. doi: 10.21037/tlcr-21-898 PMC8674609 35004256

[32] Heller JA Bhora FY Heller BJ Cohen E. Robotic-assisted thoracoscopic lung surgery: anesthetic impact and perioperative experience. Minerva Anestesiol (2018) 84:108–14. doi: 10.23736/S0375-9393.17.12168-1 28895383

[33] Ruurda JP van der Sluis PC van der Horst S van Hilllegersberg R. Robot-assisted minimally invasive esophagectomy for esophageal cancer: A systematic review. J Surg Oncol (2015) 112:257–65. doi: 10.1002/jso.23922 26390285

[34] Kingma BF Grimminger PP van der Sluis PC van Det MJ Kouwenhoven EA Chao Y-K. Worldwide techniques and outcomes in robot-assisted minimally invasive esophagectomy (RAMIE): Results from the multicenter international registry. Ann Surg (2020). doi: 10.1097/SLA.0000000000004550 33177354

[35] Park S Hwang Y Lee HJ Park IK Kim YT Kang CH. Comparison of robot-assisted esophagectomy and thoracoscopic esophagectomy in esophageal squamous cell carcinoma. J Thorac Dis (2016) 8:2853–61. doi: 10.21037/jtd.2016.10.39 PMC5107444 27867561

[36] Yang Y Li B Hua R Zhang X Jiang H Sun Y. Assessment of quality outcomes and learning curve for robot-assisted minimally invasive McKeown esophagectomy. Ann Surg Oncol (2021) 28:676–84. doi: 10.1245/s10434-020-08857-0 PMC8410169 32720046

[37] Schröder W Raptis DA Schmidt HM Gisbertz SS Moons J Asti E. Anastomotic techniques and associated morbidity in total minimally invasive transthoracic esophagectomy: Results from the EsoBenchmark database. Ann Surg (2019) 270:820–6. doi: 10.1097/SLA.0000000000003538 31634181

[38] Yang Y Zhang X Li B Li Z Sun Y Mao T. Robot-assisted esophagectomy (RAE) versus conventional minimally invasive esophagectomy (MIE) for resectable esophageal squamous cell carcinoma: protocol for a multicenter prospective randomized controlled trial (RAMIE trial, robot-assisted minimally invasive esophagectomy). BMC Cancer (2019) 19:608. doi: 10.1186/s12885-019-5799-6 31226960 PMC6587242

[39] Chao Y-K Li Z-G Wen Y-W Kim D-J Park S-Y Chang Y-L. Robotic-assisted esophagectomy vs video-assisted thoracoscopic esophagectomy (REVATE): study protocol for a randomized controlled trial. Trials (2019) 20:346. doi: 10.1186/s13063-019-3441-1 31182150 PMC6558787

[40] Tagkalos E van der Sluis PC Berlth F Poplawski A Hadzijusufovic E Lang H. Robot-assisted minimally invasive thoraco-laparoscopic esophagectomy versus minimally invasive esophagectomy for resectable esophageal adenocarcinoma, a randomized controlled trial (ROBOT-2 trial). BMC Cancer (2021) 21:1060. doi: 10.1186/s12885-021-08780-x 34565343 PMC8474742

